# Botanicals as Modulators of Neuroplasticity: Focus on BDNF

**DOI:** 10.1155/2017/5965371

**Published:** 2017-12-31

**Authors:** Enrico Sangiovanni, Paola Brivio, Mario Dell'Agli, Francesca Calabrese

**Affiliations:** Department of Pharmacological and Biomolecular Sciences, Università degli Studi di Milano, Milan, Italy

## Abstract

The involvement of brain-derived neurotrophic factor (BDNF) in different central nervous system (CNS) diseases suggests that this neurotrophin may represent an interesting and reliable therapeutic target. Accordingly, the search for new compounds, also from natural sources, able to modulate BDNF has been increasingly explored. The present review considers the literature on the effects of botanicals on BDNF. Botanicals considered were *Bacopa monnieri* (L.) Pennell, *Coffea arabica* L., *Crocus sativus* L., *Eleutherococcus senticosus* Maxim., *Camellia sinensis* (L.) Kuntze (green tea), *Ginkgo biloba* L., *Hypericum perforatum* L., *Olea europaea* L. (olive oil), *Panax ginseng* C.A. Meyer, *Rhodiola rosea* L., *Salvia miltiorrhiza* Bunge, *Vitis vinifera* L., *Withania somnifera* (L.) Dunal, and *Perilla frutescens* (L.) Britton. The effect of the active principles responsible for the efficacy of the extracts is reviewed and discussed as well. The high number of articles published (more than one hundred manuscripts for 14 botanicals) supports the growing interest in the use of natural products as BDNF modulators. The studies reported strengthen the hypothesis that botanicals may be considered useful modulators of BDNF in CNS diseases, without high side effects. Further clinical studies are mandatory to confirm botanicals as preventive agents or as useful adjuvant to the pharmacological treatment.

## 1. Introduction

One of the most complete forms of plasticity was described by Donald Hebb in 1949 who proposed an explanation for the adaptation of neurons during cognition and memory; this theory was later summarized by the famous sentence “neurons that fire together, wire together” [1]. Briefly, neuronal plasticity describes the versatility of neuronal connectivity and circuitry to which the nervous system responds and adapts to changing conditions of the body and the environment.

Among the genes involved in the modulation of neuronal activity, neurotrophic factors (NTFs), in particular the neurotrophin family of signaling proteins, play an important role in brain development [2, 3] and in adulthood modulating axonal and dendritic growth and remodeling, membrane receptor trafficking, neurotransmitter release, and synapse formation and function [4].

Brain-derived neurotrophic factor (BDNF) as well as nerve growth factor (NGF) is the most studied and best-characterized neurotrophins of the central nervous system (CNS), where they are involved in the development and maintenance of physiological brain functions. The features of the BDNF system have been extensively reviewed elsewhere [5, 6]. Briefly, in rodents, the *BDNF* gene consists of nine 5′ untranslated exons, each linked to individual promoter regions, and a 3′ coding exon (IX), which codes for the BDNF preprotein amino acid sequence [7]. Similarly, the human *Bdnf* gene is also transcribed through multiple 5′ exons spliced to a single coding exon [8]. The neurotrophin transcription is finely regulated by several intracellular signaling pathways and by different transcription factors [8–11].

Moreover, BDNF function is also highly dependent on translation and posttranslational changes. Indeed, BDNF is initially synthesized as a precursor form (proBDNF, 32 kDa) that can be cleaved into the mature neurotrophin (mBDNF, 14 kDa) or transported to the plasma membrane and released in an unprocessed manner. Upon release, the two forms of BDNF protein, as all the neurotrophins, bind with different receptors with multiple and opposite biological functions. The proBDNF binds with high-affinity p75^NTR^ leading to apoptosis, neurite retraction, and synaptic weakening and facilitating long-term depression, whereas mBDNF binds with TrkB receptors promoting cell survival, neurite extension, synaptic strengthening, and long-term potentiation (LTP) [4, 12, 13].

Alterations of NTF expression, including BDNF, are involved in the development of a variety of CNS diseases, including neurodegenerative disorders (Alzheimer's disease, Parkinson's disease, Huntington's disease, and amyotrophic lateral sclerosis) as well as psychiatric disorders (depression and schizophrenia) [14–16]. NTFs may be considered therapeutic targets, but their use has been limited so far by several, still unresolved, methodological problems aimed to guarantee their safety and efficacy [14, 15]. In particular, results from clinical studies using BDNF as a therapeutic agent have not been encouraging, possibly due to a failure of attaining relevant concentration of the trophic molecule at receptors. The two main problems seem to be related to the inability to deliver BDNF across the blood–brain barrier (BBB) and to the poor bioavailability of BDNF owing to its physiochemical properties [17].

For this reason, alternative options may be devoted to increase the endogenous content of BDNF. Accordingly, several drugs increase, indirectly, BDNF levels; however, considering the high number of nonresponder patients and the presence of serious side effects, the search for new strategies able to interfere with the mechanisms underlying CNS diseases would greatly benefit a high number of subjects. Botanicals are widely consumed all over the world as different types of products, including herbal medicinal products, plant food supplements, and functional foods. Nowadays, they are commonly used for promoting health and treating or preventing a variety of diseases even if, in most cases, clear evidence about their clinical efficacy is lacking. Emerging research provides substantial evidence to classify botanicals as modulators of markers, which are significantly altered during CNS dysfunction.

Some natural products are classified as antidepressants or anxiolytics according to the legislation of the countries in which they are sold [18, 19]. The ability of a variety of botanicals to positively modulate mood disorders and cognitive impairment resides on understanding that most of them are efficiently absorbed in humans. Recently, biologically active metabolites of botanicals able to interact with multiple targets associated with the promotion of resilience against mood disorders and cognitive impairment in response to stress have been discovered. Interventions with botanicals may benefit anxiety disorders by different mechanisms which include effects on the GABA system either via inducing ion channel transmission or through alteration of membrane structures [20]. A consistent number of botanicals, including *Ginkgo biloba* L., clinically improve cognitive impairment by ameliorating microvascular function in the brain whereas *Bacopa monnieri* (L.) Pennell has provided indications as a memory enhancer and protective agent in epilepsy [21].

**Figure 1:**
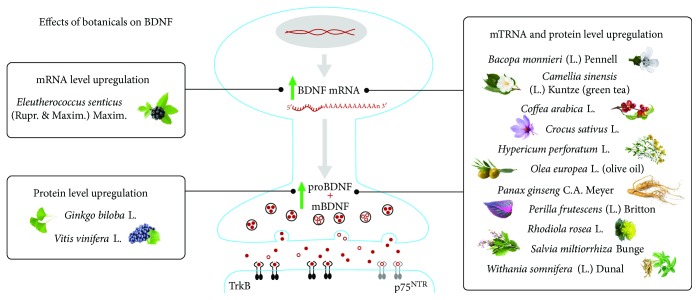
Effects of botanicals on BDNF mRNA and protein levels. The figure shows botanicals acting at transcriptional and translational levels.

The aim of the present review is to summarize the relevant literature concerning the role of botanicals as modulators of BDNF (Figure 1). Electronic literature searches were conducted in December 2016 taking into consideration also Epub articles and using Web of Science and PubMed databases. Search limit was the English language whereas no limit was applied for the year of publication. Research articles were searched for title and abstract using the following search terms: Latin name or common name or vernacular name of the plant matched with BDNF. Studies in the literature were found for the following botanicals: *Bacopa monnieri* (L.) Pennell, *Coffea arabica* L., *Crocus sativus* L., *Eleutherococcus senticosus* Maxim., *Camellia sinensis* (L.) Kuntze (green tea), *Ginkgo biloba* L., *Hypericum perforatum* L., *Olea europaea* L. (olive oil), *Panax ginseng* C.A. Meyer, *Rhodiola rosea* L., *Salvia miltiorrhiza* Bunge, *Vitis vinifera* L., *Withania somnifera* (L.) Dunal, and *Perilla frutescens* (L.) Britton.

This review emphasizes the part of the plant used, standardization of the active principles, and the protocol to manage studies in addition to the description of the behavioral test employed (Table 1). The effect of the pure compounds occurring in some plant able to modulate BDNF, such as salidroside, caffeine, epigallocatechin-3-*O*-gallate, and ginsenosides Rg1 and Rb1, will be reviewed as well whereas the effect of the pure compounds curcumin and resveratrol, which have been extensively studied as effective modulators of BDNF, will not be considered in the present review. In addition to the effect of the selected botanicals and/or their active compounds, papers describing biological effects of their association will be also considered.

## 2. *Bacopa monnieri* (L.) Pennell

*Bacopa monnieri* (L.) Pennell is a member of Scrophulariaceae traditionally used in Ayurvedic medicine for epilepsy and asthma. The best-characterized compounds occurring in the whole plant are dammarane-type triterpenoid saponins known as bacosides (mostly bacoside A), which are considered the main responsible for the biological activity [22].

### 2.1. *In Vitro* Studies

Two studies investigated the protective effect of *Bacopa monnieri* extract (BME) *in vitro*. In PC12 cells, pretreatment with a hydroalcoholic extract completely prevented the reduction of BDNF mRNA levels associated with cellular damage induced by scopolamine [23] or sodium nitroprusside [24].

### 2.2. *In Vivo* Studies

The effect of *Bacopa monnieri* was investigated, at preclinical levels, in eight studies using different animal models.

The unpredictable chronic mild stress (CMS), a well-established animal model of depression, was used to assess the ability of a compound to exert an antidepressant-like effect. Administration of BME by gavage (80 or 120 mg/kg) prevented the behavioral deficits and the reduction of 3′ UTR-long BDNF gene expression [25], as well as of the protein levels of the mature form in the hippocampus and frontal cortex of chronically stressed rats [26]. Similar effects were observed after treatment with the tricyclic antidepressant drug imipramine [25–27].

The cognition-enhancing properties of *Bacopa monnieri* were investigated in the scopolamine rodent model of “cholinergic amnesia” and in the olfactory bulbectomy (OBX), a model of cognitive and emotional dysfunction typical of neurodegenerative pathologies such as Alzheimer's disease [28].

Interestingly, chronic treatment (62 days) with an alcoholic BME (50 mg/kg in the drinking water) ameliorated the memory disturbance and completely normalized the reduction of hippocampal BDNF mRNA levels due to the OBX [29].

Moreover, 1 week of oral (os) treatment with the hydroalcoholic BME (10, 20, and 40 mg/kg) dose-dependently prevented the memory deficits induced by scopolamine and normalized the reduction of BDNF mRNA levels in the rat hippocampus [30]. A similar effect was found in young mice at postnatal day (PND) 30 [31] treated with CDRI-08 (BME standardized in bacoside A) (3 mg/kg, i.p., 7 days) before and after scopolamine injection. Both the protocols attenuated the decrease in proBDNF protein levels in the mouse cerebrum, caused by scopolamine administration. Furthermore, CDRI-08 per se induced an increase in BDNF gene and protein expression [31].

In a recent study, administration of CDRI-08 (80 mg/kg, i.p., 2 weeks) significantly increased the mRNA and the protein levels of proBDNF in the hippocampus of young rats (PND 32). Interestingly, this effect paralleled the upregulation of the unmethylated CpG islands 1 and improved the object recognition memory [32]. Using the same regime of treatment, CDRI-08 facilitated memory acquisition in the fear-conditioning paradigm and increased the expression of BDNF exon IV transcript in the hippocampus of PND 30 rats [33].

On this basis, even if clinical studies are needed, preclinical results indicate that *Bacopa monnieri* extract administration modulates a BDNF effect that may underline its ability as an antidepressant and procognitive agent.

## 3. *Coffea arabica* L.

The coffee plant, a woody perennial tree growing at higher altitudes, belongs to the family of Rubiaceae. Although beans are particularly rich in caffeine, other constituents are present in a considerable amount, including tocopherols and caffeic acid derivatives, such as chlorogenic acid.

In the literature are present studies reporting the effect of caffeine on BDNF, whereas the effect of a *Coffea arabica* extract from fruits was investigated only in one clinical study.

### 3.1. *In Vitro* Studies

Three studies investigated the *in vitro* effect of caffeine on BDNF. In particular, caffeine upregulated the BDNF protein levels in mouse hippocampal slices (100 *μ*M for 5 minutes) [34], increased the BDNF release in hippocampal neurons [35], and efficiently stimulated the BDNF isoform I and IV expression in the presence of KCl (10 mM) in cortical neurons [36].

### 3.2. *In Vivo* Studies

15 studies investigated the effect of caffeine on BDNF *in vivo*. Treatment of zebrafish embryos with caffeine (100 *μ*M) increased the BDNF mRNA levels specifically after 48 and 72 hours postfertilization [37].

Caffeine administration in naïve rats, during adulthood, counteracted the negative effect exerted by its intake in early life by increasing the protein levels of mBDNF [38]. Differently, administration of caffeine (1.0 g/L in drinking water) two weeks before mating, during pregnancy, and up to embryonic days 18–20 (E18 or E20) caused a decrease in BDNF protein levels in the whole cortex until E18, while an increase was found at E20 [39].

Caffeine intake during adolescence (from PND 28 to PND 53) by drinking water decreased both proBDNF and mBDNF in the hippocampus at 1.0 mg/mL, while an increase was found in the cerebral cortex at 0.3 and 1.0 mg/mL [40]. Interestingly, caffeine at 0.1 or 0.3 mg/mL improved recognition memory while the highest dose impaired the nonassociative memory [40].

During adulthood to old age, 30 consecutive days of free access to drinking water containing 1 mg/mL of caffeine solution reduced age-related memory impairment and increased proBDNF in the hippocampus of young adult (3 months old) and middle-aged rats (12 months old); the treatment also prevented the age-related increase in the mature form in older rats [41].

Accordingly, prolonged treatment (12 months) with caffeine solution (1 mg/mL in drinking water) in 6-month-old mice counteracted the increase in mBDNF in the hippocampus of aged animals and prevented the age-associated memory decline [42]. Moreover, 4 consecutive days of caffeine treatment (10 mg/kg, i.p.) increased the protein levels of mBDNF in the same brain region and improved the performance in the object recognition task in adult mice [43].

In the hippocampus of a mouse model of Alzheimer's disease, induced by AlCl_3_, cotreatment with caffeine (1.5 mg/day by gavage) partially prevented the decrease in BDNF gene expression, while the pretreatment completely normalized the impairment [44]. Accordingly, chronic caffeine treatment (0.75 mg/day or 1.5 mg/day for 8 weeks) dose-dependently increased the mBDNF protein levels in the hippocampus of APP/PS1 (A*β* precursor protein/presenelin-1) double transgenic mice, another model of Alzheimer's disease, and reversed the memory impairment observed in the Morris water maze (MWM) test [45].

Chronic caffeine treatment (0.33 mg/L in drinking water) during 4 weeks of psychological stress (intruder model) restored the reduced BDNF protein levels found in the stressed group [46]. The i.p. injection of caffeine once a week was enough to normalize the deficit of BDNF protein levels induced by a high-fat diet. At behavioral level, caffeine fully prevented the diet-induced impairment and restored the spatial memory observed in control animals. Neither diet nor caffeine treatment affected motor activity [47].

Since sleep is a critical factor in memory consolidation and neural plasticity [48], the effect of the chronic caffeine treatment on sleep loss was investigated. Oral administration of caffeine (60 mg/kg) or the psychostimulant modafinil (100 mg/kg), at the onset of the light phase during 48 hours of sleep deprivation (SD), restored the normal levels of cell proliferation improving BDNF expression in the dentate gyrus [49]. Accordingly, 4 weeks of caffeine treatment in drinking water (0.3 g/L) prevented the SD-induced decrease in neurotrophin levels in the dentate gyrus and in the cornu ammonis-1 (CA1) of the hippocampus [50, 51] and alleviated the impairment in the spatial long-term memory observed in sleep-deprived rats, also through the modulation of BDNF protein levels [52].

In summary, caffeine affects BDNF protein levels with a specific temporal and dose profile in normal animals. While administration during adulthood or old age increased BDNF, caffeine intake at high doses in early life downregulated the neurotrophin concentration.

Moreover, even if few studies investigated the efficacy of caffeine in animal models of pathology, they provide promising results.

At behavioral levels, caffeine was evaluated as a cognitive enhancer with positive effects.

### 3.3. Clinical Studies

The clinical study by Reyes-Izquierdo et al. [53] investigated the effect of three different coffee fruit extracts (100 mg dose per os) on BDNF plasma levels in healthy subjects. Coffee fruit concentrate powder (WCFC) (0.7% caffeine) but neither green coffee caffeine (N677) (72.8% caffeine) nor green coffee bean extract (N625) (2% caffeine) increased the level of BDNF in blood suggesting that the effect of WFCF might be related to the amount of procyanidins rather than to caffeine [53].

## 4. *Crocus sativus* L.

*Crocus sativus* L. belongs to the Iridaceae family; stigmas are commonly known as saffron and are widely cultivated in Iran and used in modern and traditional medicines. The color of saffron is mostly due to the carotenoid named crocin, which is considered among the active principles mostly responsible for neuroprotective activity [54].

### 4.1. *In Vivo* Studies

Two *in vivo* studies investigated the effect of *Crocus sativus* on BDNF expression. Crocin administration (12.5 mg/kg, i.p.) for 21 days to naïve male Wistar rats exerted an antidepressant effect and significantly increased the transcription levels of BDNF in the hippocampus [55]. Similarly, chronic treatment with *C. sativus* aqueous extract (40, 80, or 160 mg/kg/day, i.p.), enhanced the gene and protein levels of BDNF in the rat hippocampus. Moreover, at 40 and 160 mg/kg/day, an antidepressant activity was also observed. Similar results were obtained following imipramine injection (10 mg/kg) [56].

## 5. *Eleutherococcus senticosus* (Rupr. & Maxim.) Maxim.

*Eleutherococcus senticosus* Maxim. or *Acanthopanax senticosus* Harms, also called “Siberian ginseng,” is a small shrub from the Araliaceae family. *Eleutherococcus* consists of the whole or cut dried roots of the plant containing lignans, phenylpropanoids, and dicaffeoylquinic acids [57].

### 5.1. *In Vitro* Studies

One paper investigated *in vitro* the effect of *Eleutherococcus senticosus* on BDNF. A commercial dry aqueous extract of *Acanthopanax senticosus* stem bark (ASE) normalized the reduction of BDNF mRNA levels produced by the administration of corticosterone (200 *μ*M) for 24 h in PC12 cells. Different concentrations of ASE (100, 200, and 400 *μ*g/mL) significantly increased the mRNA expression of the neurotrophin in a concentration-dependent fashion [58].

Unfortunately, no *in vivo* or clinical studies are reported in the literature on the modulation of BDNF by *E. senticosus*, and no clear-cut conclusions can be drawn.

## 6. *Ginkgo biloba* L.

*Ginkgo biloba* is an ancient Chinese tree belonging to the family of Ginkgoaceae, cultivated for its health-promoting properties. Although both leaves and seeds are currently used as herbal medicine in China, in many countries, leaves are considered the unique source of active principles and dried green leaves are used for supplying pharmaceutical formulations or extracts as ingredients of food supplements. *Ginkgo biloba* and its constituents were evaluated on BDNF in three *in vitro*, eight *in vivo*, and one clinical studies.

### 6.1. *In Vitro* Studies

*Ginkgo biloba* leaf extract (EGb761, 100 *μ*g/mL) restored the levels of BDNF protein (both pro and mature form) in cells stimulated with appropriate medium able to induce amyloid *β*-peptide A*β* expression.

Administration of individual EGb761 constituents, namely, ginkgolides A (GA), B (GB), C (GC), and J (GJ) and 10 *μ*g/mL bilobalide, increased the levels of BDNF by following a similar pattern [59].

Accordingly, flavonol-enriched extract containing quercetin, kaempferol, and isorhamnetin (50 *μ*g/mL) significantly restored BDNF protein expression in double transgenic APP/PS1 primary neurons [60].

Moreover, 100 *μ*g/mL of YY162, a patented formula consisting of terpenoid-strengthened *Ginkgo biloba* and ginsenoside Rg3, prevented the reduction of BDNF protein levels induced by 48 h of Aroclor 1254 in SH-SY5Y neuroblastoma cell line [61].

### 6.2. *In Vivo* Studies

*Ginkgo* flavonols (50 mg/kg, per os, daily for 4 months) significantly normalized the deficit of BDNF protein levels in the hippocampus of transgenic APP/PS1 mice and improved spatial learning similar to the administration of the antidepressant SSRI (serotonin selective reuptake inhibitor) fluoxetine (10 mg/kg), while exerting an antidepressant effect on wild-type animals [60].

YY162 (200 mg/kg, per os, from PND 21 to PND 35) significantly attenuated the reduction of BDNF protein in the prefrontal cortex and ameliorated the ADHD- (attention deficit hyperactivity disorder-) like behavioral phenotype induced by Aroclor 1254 [61].

Intravenous (i.v.) injections of EGb761 (45 mg/kg), just before ischemia-reperfusion, induced a significant increase in BDNF positive neurons in the hippocampus with respect to the control group; the treatment significantly reduced the behavior grade measured by a postural reflex test at 24 h after reperfusion. The effect exerted by EGb761 was comparable to that exerted by the antihypertensive nimodipine (2 mg/kg) [62].

Chronic treatment with EGb761 (100 mg/kg/day via oral gavage for 30 days) increased the BDNF levels in plasma of both young and aged (18 months) rats, but the effect was not statistically significant; on the opposite, in the aged female group, treatment significantly increased the number of platform crossings in the aged female group in the open field test (OFT) [63].

Pretreatment with EGb761 (100 or 150 mg/kg/day, per os for 10 days) significantly inhibited the reduction of hippocampal BDNF protein due to LPS (lipopolysaccharide) injections (0.83 mg/kg, i.p.) and showed an antidepressant effect [64]. Furthermore, EGb761 treatment (50 mg/kg/day, by oral gavage for 5 weeks) normalized the reduction of BDNF protein levels induced by the first-generation antipsychotic haloperidol injection (2 mg/kg/day, i.p., 5 weeks) in the prefrontal cortex, striatum, substantia nigra, and globus pallidus and reduced the vacuous chewing movement scores over the withdrawal period [65]. Finally, 28 days of treatment with EGb761 (40 mg/kg) increased the expression of BDNF and explored the behavior in stressed rats. The effect was comparable to that of the SNRI (serotonin noradrenaline reuptake inhibitor) antidepressant venlafaxine (15 mg/kg) [66]. Administration of bilobalide (10 mg/kg, i.p.) for 10 days enhanced the hippocampal protein levels in normal mice more efficiently than that of fluoxetine (10 mg/kg) [67].

Taken together, these preclinical results suggest that *Ginkgo biloba* L. administration may be efficacious in restoring BDNF in pathologies characterized by neurotrophin deficits. The main problem is that studies take into consideration different animal models mimicking different kinds of diseases, from Alzheimer's disease to stroke, thus making further results mandatory to confirm the supposed effect on BDNF.

### 6.3. Clinical Studies

In the unique clinical study, one hundred fifty-seven patients affected by tardive dyskinesia (TD) associated with long-term neuroleptic treatment were randomized to either EGb761 80 mg three times a day or placebo treatment. EGb761 significantly increased the BDNF protein plasma levels compared with placebo at week 12 in TD patients [68].

## 7. Green Tea (*Camellia sinensis* (L.) Kuntze)

Tea obtained from the dried leaves of *Camellia sinensis* (L.) Kuntze (Theaceae) is one of the most widely consumed beverages in the world. Green tea (GT) contains many bioactive compounds including amino acids (i.e., L-theanine), flavonoids (i.e., catechins), and their derivatives, which may constitute up to 30% of the dried weight [69].

### 7.1. *In Vitro* Studies

The potential neuroprotective effect of some constituents of green tea leaves, including catechins, was investigated in two *in vitro* studies.

L-Theanine pretreatment (500 *μ*M) exerted a protective effect by significantly attenuating the downregulation of BDNF protein due to the treatment with two disease-related neurotoxicants (rotenone and dieldrin) in the human cell line SH-SY5Y [70].

Moreover, pretreatment with GT catechins, such as epicatechin (EC) and (+)-catechin, prevented the reduction of mBDNF and the increase in the precursor form induced by the toxic HIV (human immunodeficiency virus) protein Tat [71].

### 7.2. *In Vivo* Studies

10 papers investigated the effects of GT on BDNF. Chronic administration of L-theanine at different doses (0.2, 0.4, and 10 mg/kg, i.p.) exerted an antidepressant activity and upregulated the protein levels of BDNF in the hippocampus, but not in the cortex of adult mice [72]. Moreover, daily consumption of the flavonol (−)-EC (4 mg/day in water for 14 weeks, ad libitum) in adult mice led to an anxiolytic-like effect and increased the pro and mBDNF levels in the hippocampus, while no effect was found in the cortex [73].

The effect of theanine administration during development was evaluated on rat pups receiving 0.3% theanine (through lactation before weaning and then directly by drinking water) showing increased exploratory activity and enhanced object recognition memory and levels of mBDNF protein in the hippocampus [74].

Assuncao et al. demonstrated that the decrease in BDNF protein levels in the rat hippocampus, associated with aging, was prevented by drinking GT-infused drink as the only drink available from 12 to 19 months of age [75]. Similarly, catechins (0.05% and 0.1%) mixed with drinking water for 6 months improved age-related spatial learning and memory decline of 14-month-old female mice and upregulated the hippocampal mature form of BDNF to levels comparable to those observed in young animals [76].

On the contrary, the addition of epigallocatechin-3-*O*-gallate (EGCG) (182 mg/kg/day) and *β*-alanine (417 mg/kg/day) to the diet for 4 months did not improve memory and did not alter the mRNA expression of BDNF in the hippocampus of 19-month-old male mice [77].

The GT effect has been studied in different animal models of learning impairments. Administration (1 g in 100 mL water for 5 min at 100°C, corresponding to 0.6–1 mg EGCG per day), from gestation to adulthood, corrected the lower BDNF mRNA levels in the hippocampus of mice overexpressing *DYRK1A* (dual-specificity tyrosine phosphorylation-regulated kinase 1A) but did not affect the performance in memory tasks [78].

In senescence-accelerated mice-prone 8 (SAMP8), a model characterized by the early onset of learning and memory deficits along with overproduction of soluble amyloid peptide in the brain, the chronic treatment with green tea catechins (GTC) (0.05% and 0.1% in drinking water for 6 months) restored the reduction of mBDNF levels in the hippocampus and prevented the learning impairment of SAMP8 mice [79]. Finally, 4 months of EC administration (50 mg/kg daily in drinking water) normalized the low levels of BDNF protein in the hippocampus of 8-month-old APP/PS1 mice, without affecting escape latency in MWM [80].

Teasaponin (10 mg/kg, i.p. for 21 days) rescued the upregulation of BDNF induced by the adipocyte-secreted hormone leptin in the prefrontal cortex of high-fat diet-fed mice. In addition, teasaponin (20 or 40 *μ*M) reversed the effect of palmitic acid on the alteration induced by leptin in cultured cortical neurons [81].

To summarize, these studies provide robust evidence regarding the role of green tea as a modulator of BDNF and in improving cognitive performance at preclinical levels.

## 8. *Hypericum perforatum* L.

*Hypericum perforatum* L. (HYP), known as St John's wort, is a plant belonging to the family of Hypericaceae. Flowering aerial parts are used in many countries for their antidepressant activity, mostly ascribed to the active principles hyperforin, hypericin, and pseudohypericin [18, 57].

### 8.1. *In Vivo* Studies

The antidepressant activity of *Hypericum perforatum* was investigated at preclinical level in two *in vivo* studies using the CMS animal model. The chronic treatment with hydroalcoholic extract of HYP (350 mg/kg per os, 21 days) normalized the reduction of mRNA expression of BDNF found in the hippocampus of stressed mice [82]. On the contrary, Butterweck et al. showed that the chronic administration of HYP methanolic extract (500 mg/kg, per os) did not prevent the stress-induced decrease in BDNF mRNA levels in the rat hippocampus, produced by the immobilization stress protocol (2 h once a day for 7 days) [83]. The different outcomes between these two preclinical studies could be due to the type of stressors or the protocol of treatment used.

### 8.2. Clinical Studies

In one large cohort of subjects, chronic HYP treatment restored the normal protein concentration of BDNF in the serum of depressed patients. This normalization was limited to HYP and serotonin reuptake inhibitors, whereas other classes of antidepressants, including the tricyclics and the noradrenergic and specific serotoninergic antidepressant, were ineffective. The limitation of this study is that the dose of the different drugs used is not clearly indicated [84].

## 9. Olive Oil (*Olea europaea* L.)

Olive oil is the main source of fat in the Mediterranean-style diet. Health benefit of olive oil consumption has been in part ascribed to minor phenol components (i.e., oleuropein, ligstroside aglycones, and hydroxytyrosol (HT)) whose composition varies qualitatively and quantitatively depending on the stage of fruit ripeness or the region of cultivation [85, 86].

### 9.1. *In Vivo* Studies

Five animal studies were performed to test the effect of olive oil components on BDNF. The administration of a mixture of olive oil polyphenols extracted from the olive residues (pomace) (10 mg/kg, i.p., 10 days) significantly increased the BDNF protein levels in the hippocampus and in the olfactory lobes, while decreasing the neurotrophin in the frontal cortex [87]. Moreover, this regime of treatment did not affect pain sensitivity in the hot-plate test or stress response in the FST in naïve animals [87].

Differently, if the blend of polyphenols was extracted from olive leaves (20 mg/kg, i.p. for 15 days), BDNF protein levels were downregulated in the hippocampus and striatum and upregulated in the olfactory lobes. Polyphenol administration significantly increased the concentration of BDNF protein in the mouse serum [88]. Interestingly, a diet enriched in olive oil components during prenatal life until weaning induced, at adulthood, an upregulation of the mRNA levels of the total BDNF and of the isoforms IV and VI in the prefrontal cortex but not in the hippocampus [89].

Before mating, the treatment with HT (10 or 50 mg/kg/day by gavage for 2 weeks), one of the most bioactive phenolic compound in olive oil, prevented the significant decrease in proBDNF and mBDNF due to prenatal stress exposure in male offspring and improved cognitive functions [90].

In Alzheimer's disease mouse model, HT chronic treatment (10 mg/day by gavage for 14 days) attenuated the spatio-cognitive deficits and normalized the hippocampal BDNF mRNA levels [91].

### 9.2. Clinical Studies

The effect of olive oil was investigated in two clinical studies. Taking Mediterranean diet supplemented with olive oil for 3 years did not alter the plasma BDNF protein levels in normal subjects [92]. Moreover, the administration of a blend of olive polyphenols (a tablet containing a total of 50 mg/day) extracted from the olive pomace and containing mostly HT and oleuropein for 15 consecutive days in alcoholic patients undergoing withdrawal induced a transient decrease in mBDNF protein levels in the serum after 3 days of treatment [93].

Even if not so many, *in vivo* studies provide positive results, while the effect in clinical studies (only 2) appears inconsistent or negligible.

## 10. *Panax ginseng* C.A. Meyer

Ginseng radix consists of the whole or cut dried root of *Panax ginseng* C.A. Meyer and contains not less than 0.4% of the sum of ginsenosides Rg1 (Rg1) and Rb1 (Rb1). Ginsenosides are triterpenoid saponins which are the main responsible for the biological activities of ginseng extracts [57].

### 10.1. *In Vitro* Studies

Five studies were performed *in vitro* to test the effect of ginseng or ginsenosides on BDNF. Rg1 treatment upregulated the mRNA expression and protein secretion of BDNF in primary cultured olfactory cells (Rg1, 40 *μ*g/mL for 72 h) [94] and in Schwann cells (Rg1, 50 *μ*M for 24 h) challenged with 0.2% H_2_O_2_ for 4 h [95]. The beneficial effect was also found in different “pathological conditions.” Indeed, pretreatment of rat brain slices with Rg1 at different concentrations (60, 120, and 240 *μ*M for 2 h), before okadaic acid administration, increased the BDNF protein expression in a dose-dependent fashion [96].

Red ginseng extract (RGE) (0.01–1.0 mg/mL applied for 1 h) dose-dependently increased the BDNF protein expression in primary cultures of rat hippocampal neurons exposed for 48 h to 100 *μ*M kainic acid [97]. BDNF protein levels were significantly increased in PC12 cells subjected to oxygen glucose deprivation/reperfusion (OGD/R) for 4 h by ginsenoside Rd (Rd) at 50 and 100 *μ*M [98].

### 10.2. *In Vivo* Studies

A total number of 25 studies were performed *in vivo*, mostly on pure ginsenosides.

Deficits induced at protein levels by the exposure to CMS were normalized by concomitant treatment with ginseng standardized in the saponin content (GTS) (50 and 100 mg/kg) [99] or Rg1 (2.5, 5, 10, and 20 mg/kg, i.p.) [100] in the hippocampus and with ginsenoside Rb3 (Rb3) (30, 75, and 150 mg/kg, intragastrically) both in the prefrontal cortex and in the hippocampus [101]. At 40 mg/kg (i.p.), the effect, for Rg1, was also found in the lateral amygdala [102, 103]. Rg1 corrected the alteration found at translational level [100] similar to fluoxetine (10 mg/kg) [104, 105] and imipramine [100]. Moreover, all the compounds reverted the behavioral phenotype associated with this model [99–103].

A comparable effect was found after chronic restraint stress with Re (50 mg/kg) and fluoxetine (10 mg/kg) at transcriptional level in the hippocampus [106], while Rg1 (10 mg/kg) prevented the reduction of mBDNF in the prefrontal cortex [107]. Re produced an antidepressant and anxiolytic effect [106], whereas Rg1 improved learning and memory [107].

The decreased expression of BDNF mRNA, found after the single prolonged stress, was significantly restored to normal level by chronic treatment with Rb1 (10 or 30 mg/kg, i.p., 14 days) or fluoxetine (10 mg/kg). Rb1 at 30 mg/kg normalized the percentage of time spent in the open arms in the elevated plus maze [104]. Rb1 (10 mg/kg), administered 30 min before acute immobilization stress, significantly inhibited the stress-mediated decline in BDNF mRNA level [108].

Accordingly, GTS (25 or 50 mg/kg/day), similar to fluoxetine (10 mg/kg), significantly upregulated the mRNA and protein levels of BDNF in the hippocampus of corticosterone-treated mice (20 mg/kg, once a day for 22 days) but not in that of normal animals and produced an antidepressant effect [105].

*Panax ginseng* extract or pure compounds exerted a positive effect also on the scopolamine animal model. Indeed, wild ginseng (WG) roots (200 mg/kg, i.p.) normalized the mRNA level of BDNF in the rat hippocampus of the scopolamine-treated group, as well as reducing the escape latency in the MWM test [109]. Accordingly, pretreatment with ginsenosides Rg5 (Rg5) and Rh3 (Rh3) (5, 10, and 20 mg/kg, per os) inhibited the reduction of mBDNF protein expression induced by scopolamine injection (1 mg/kg, i.p.) and reduced the latency time in MWM. The protective effect of Rh3 (5 and 10 mg/kg) on memory deficit was more potent than that of Rh5 and comparable with that of the acetyl cholinesterase inhibitor donepezil (5 mg/kg) used in the treatment of Alzheimer's disease [110].

Oral administration of Rg5 (5, 10, and 20 mg/kg) or donepezil (3 mg/kg) prevented the reduction of mBDNF induced by streptozotocin (STZ) (3 mg/kg intracerebroventricular administration) and enhanced the memory retention, the mean latency time, and the path length with respect to the STZ group in the MWM test [111].

Rd (10, 20, 40, and 80 mg/kg/day, i.p.) prevented the reduction of BDNF expression in both the cerebral cortex and lumbar spinal cord in an animal model of encephalomyelitis [112].

Rg1 (2.5, 5.0, and 10 mg/kg, i.p.) significantly increased, in a dose-dependent manner, the mBDNF protein level in SAMP8 mice and ameliorated the cognitive impairments observed in 9-month-old mice [113].

Similarly, chronic treatment with Rg1 (1 mg/kg or 10 mg/kg, i.p. for 30 days) significantly enhanced the mBDNF expression in the hippocampal homogenate of middle-aged rats. Also, proBDNF was upregulated, but the effect was significant only for the 10 mg/kg-treated group. Furthermore, Rg1 administration significantly improved the memory in the fear-conditioning task [114].

The protective effect of Rg1 on memory performance and synaptic plasticity was assessed in a transgenic AD model constructed by overexpressing APP and PS1. The injection of 10 mg/kg Rg1 for 30 days (i.p.) upregulated the BDNF protein levels and ameliorated the memory in mice [115].

Moreover, in adult male rats, Rb1 infusion (40 mg/kg) significantly increased the BDNF protein expression from 3 h to 10 days after middle cerebral artery reperfusion, with a peak at 3 days [116]. On the opposite, treatment with Rb1 (7.5 mg/mL) by intragastric administration for three days, three times a day (12.5 mL/kg weight) before transient middle cerebral artery occlusion, did not prevent the increased levels of BDNF due to the damage. Indeed, BDNF protein levels were higher in the Rb1 group compared to the untreated animals [117].

Five- to 7-week-old male C57BL/6J mice were treated with Rd (10 or 30 mg/kg, i.p. for 21 days) fifteen days after bilateral carotid artery stenosis that induced chronic cerebral hypoperfusion (CCH). The dramatic decrease in BDNF protein and mRNA levels observed in the CCH model was reversed by Rd administration that also improved the memory task performance [118].

YY162 significantly reduced the BDNF protein decline in the prefrontal cortex and improved the ADHD behavioral phenotype [61].

Pretreatment for 3 days with Rg1 (10 or 30 mg/kg, i.p.) or the antibiotic minocycline (30 mg/kg, i.p.) significantly normalized the BDNF mRNA levels altered by a central injection of LPS (5 *μ*g in 5 *μ*L saline) in the cortex but not in the hippocampus.

Rg1 administration at both low and high doses alleviated the anorexic symptoms and increased the sucrose preference [119].

The effect of ginsenoside administration on naïve animals is conflicting. Indeed, injections of Rb1 5 mg/kg/day in 0.2 cc saline i.p. for 4 days did not modulate the BDNF mRNA levels in the hippocampus [120], while ginsenoside Rh1 (10 mg/kg/day for 3 months) significantly upregulated the BDNF protein levels in the hippocampus with respect to the control group. The Rh1-treated group (5 and 10 mg/kg) significantly promoted the spatial learning ability in the MWM test [121]. Finally, administration of gintonin (50 mg/kg per os, 7 days), a mixture of glycolipoproteins from *Panax ginseng*, significantly increased the BDNF protein levels [122].

Taken together, reviewed studies suggest that pure ginsenosides are effective modulators of neuroplasticity. The main criticism is the heterogeneity of the studies that evaluate the effect of each ginsenoside at a time.

## 11. *Perilla frutescens* (L.) Britton

*Perilla frutescens* (L.) Britton, also called *zi-su* in Chinese, is an annual herb belonging to the Lamiaceae family; stems, leaves, and seeds are widely used in traditional Chinese medicine or as food ingredients.

### 11.1. *In Vivo* Studies

Five papers investigated the effect of *Perilla frutescens* on *in vivo* models. Administration of essential oil from commercial *Perilla* leaf (EOPL) for 4 weeks, at 3 or 6 mg/kg, normalized the BDNF gene expression, while only the highest dose was effective at protein levels in the hippocampus of chronically stressed mice. Conversely, 3 weeks of EOPL was not enough to correct the molecular deficit observed. Moreover, EOPL produced an antidepressant-like effect in the sucrose preference test after 3 and 4 weeks at both concentrations and in the FST at 6 mg/kg after 3-week treatment or at 3 mg/kg and 6 mg/kg after 4-week treatment, while no effect was observed on the locomotor activity. Interestingly, a similar effect was found in administering 20 mg/kg fluoxetine [123].

Male mice subjected to dietary restriction of *α*-linolenic acid (ALA) were fed with a diet supplemented with *Perilla* oil (5%) for 8 weeks. ALA restriction lowered the BDNF levels in the striatum, and *Perilla* oil significantly increased the BDNF protein levels [124, 125].

Six weeks of *Perilla* seed oil administration at 4% (*w*/*w*) in the diet to naïve Sprague-Dawley rats significantly upregulated the concentration of BDNF in the prefrontal cortex, while the immobility times were significantly shorter in the FST. A similar effect was observed after i.p. injection of imipramine (30 mg/kg) [126].

In a recent study, chronic treatment with *Perilla* oil (500 mg/kg/day by gastric gavage) normalized the decrease in BDNF protein levels in an animal model of Alzheimer's disease. The effect paralleled the anxiolytic-like effect and improved the cognitive performance measured in both the novel object recognition test and the MWM test [127].

On these bases, the few studies present in literature are encouraging, but other demonstrations are mandatory to draw clear-cut conclusions.

## 12. *Rhodiola rosea* L.

*Rhodiola rosea* L. (Crassulaceae) has a long history of use as a medicinal plant in several traditional medicines. *Rhodiola* root and rhizome increase the organism's resistance to physical, chemical, and biological stressors; the effect is mostly due to the active principle salidroside (SA, syn. rhodioloside) [128].

### 12.1. *In Vitro* Studies

One *in vitro* study evaluated the effect of SA on BDNF demonstrating that the pure compound induced mesenchymal stem cells to differentiate into dopaminergic neurons. Moreover, SA treatment (100 *μ*g/mL) for 1–6 days significantly increased the BDNF mRNA levels while at 12 days, an opposite effect was found. Differently, the effect on the BDNF protein levels was more long lasting since it was still present after 12 days [129].

### 12.2. *In Vivo* Studies

*In vivo*, the treatment for 5 days (12 and 24 mg/kg, per os) with SA or fluoxetine prevented the development of the depression-like behavior and of the downregulation of BDNF protein levels in the hippocampus induced by a single injection of LPS [130].

## 13. *Salvia miltiorrhiza* Bunge

*Salvia miltiorrhiza* Bunge (Lamiaceae), also known as red sage, is a perennial plant; root and rhizome are widely used in China for the treatment of cardiovascular and cerebrovascular diseases [131].

### 13.1. *In Vitro* Studies

Two *in vitro* studies evaluated the effect of salvianolic acid B (SalB). Treatment with SalB (20 *μ*g/mL, for 24 h) significantly increased the level of BDNF protein in bone marrow-derived neural stem cells [132].

Furthermore, SMND-309 (5, 10, and 20 *μ*M for 24 h), the metabolite produced in the brain and heart of rats after SalB oral administration, restored the mBDNF protein expression in the human neuroblastoma cell line SH-SY5Y subjected for 2 h to OGD/R [133].

### 13.2. *In Vivo* Studies

*Salvia miltiorrhiza* extracts and pure compounds were also investigated in five *in vivo* studies. Hippocampal BDNF immunoreactivity was markedly decreased by the injection of the A*β*_25–35_ peptide in mice, and subchronic treatment with SalB (10 mg/kg, 7 days) reversed this reduction [134]. A similar effect was observed after oral treatment for 14 days (0.81 and 0.405 g/kg) with the formulation named Compound Danshen Tablet (CDT) (*Salvia miltiorrhiza*, *Panax notoginseng*, and borneol in a ratio 450 : 141 : 8). Indeed, CDT administered to mice at 0.81 g/kg or 0.405 g/kg normalized the hippocampal BDNF mRNA levels and improved the cognitive performance, while the lowest dose was effective also at the protein level [135].

Pretreatment with tanshinone I (10 mg/kg, i.p. for 3 days), a lipophilic diterpenoid occurring in the radix of *Salvia miltiorrhiza*, administered 5 minutes before ischemia-reperfusion by bilateral common carotid artery occlusion, corrected the reduced BDNF immunoreactivity in the CA1 of ischemic Mongolian gerbils [136]. Similarly, salvianolic acid A (100 *μ*g/kg) administered intravenously 2 h after middle cerebral artery occlusion/reperfusion significantly reversed the protein levels of mBDNF in the ipsilateral ischemic brain hemisphere. Furthermore, the treatment significantly improved the reduction in tracking distance induced by stroke injury [137].

Salvianolate lyophilized injection (10.5, 21, and 42 mg/kg) ameliorated the deficits observed in diabetic rats after stroke normalizing the protein level of the mature form of BDNF [138].

## 14. *Vitis vinifera* L. (Red Wine)

*Vitis vinifera* L. is a plant belonging to the family of Vitaceae, native to the Mediterranean region and widely used to make wine or juice.

### 14.1. *In Vivo* Studies

Two *in vivo* studies were performed on male rats. Grape powder (including that of fresh red, green, and blue-black California grapes, seeded and seedless varieties) dissolved in tap water (15 g/L) for 3 weeks attenuated the reduction of BDNF protein levels induced by the oxidative stress mediator L-buthionine-(S,R)-sulfoximine in the amygdala, hippocampus, and cortex [139] or due to the exposure to a single prolonged stress [140]. Moreover, grape intake exerted an anxiolytic effect and a positive effect in the memory test [139, 140]. In addition to grape studies, few interesting papers investigated BDNF modulations by red wine or ethanol starting from 60 days before pregnancy up to pup weaning. Adult animals exposed to only ethanol showed disrupted levels of BDNF in several brain areas, including the hippocampus, and altered cognition and emotional behavior. Conversely, mice exposed to red wine had no changes in the behavior but a decrease in hippocampal BDNF [141]. Another study investigated BDNF changes in old male mice following perinatal exposure to ethanol or red wine at the same ethanol concentration. The study demonstrates that ethanol alone is able to increase BDNF levels in limbic areas, whereas, in mice exposed to red wine, BDNF levels were comparable to those of control thus allowing one to hypothesize a protective role of wine polyphenols against the damaging effect of ethanol alone [142].

### 14.2. Clinical Studies

One clinical study was performed on 25 healthy fasted subjects treated with a single dose of grape seed extract (N31, 72% polyphenols). The participants were 18–55 years old and had a body mass index between 18.0 and 25.0 kg/m^2^. N31 increased the BDNF levels in plasma by 30% with respect to the baseline, although the effect was not statistically significant [53].

## 15. *Withania somnifera* (L.) Dunal

*Withania somnifera* (L.) Dunal, also called Ashwagandha or Indian ginseng (Solanaceae), is a traditional Ayurvedic remedy reputed to be useful as an antistress and memory enhancer [143].

### 15.1. *In Vivo* Studies

Two papers investigated the effect of *Withania somnifera* on *in vivo* models. Pretreatment with an alcoholic extract of Ashwagandha leaves (100 mg, 200 mg, and 300 mg/kg for 7 days) significantly prevented the effects due to the scopolamine treatment (3 mg/kg) such as the reduction of the mRNA expression of BDNF transcript variant-1 and of proBDNF and mBDNF protein expression at all the concentrations tested. On the contrary, posttreatment at 200 mg/kg was ineffective [144].

Withanolide-enriched extract from the *Withania somnifera* root (methanol–water 25 : 75, *v*/*v*) was evaluated on induced hypobaric hypoxia in rats. Animals fed before and after hypobaric hypoxia with 200 mg/kg of the extract showed an increased expression of BDNF and a significant decrease in latency and path length in the MWM test [145].

## 16. Conclusions

**Figure 2:**
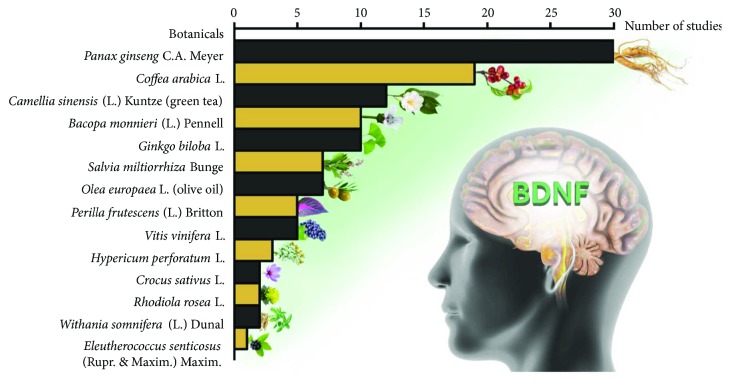
Number of studies investigating BDNF in the central nervous system for each botanical or active principle.

In this review, we provide an upgrade of the current literature on the ability of some well-known botanicals to modulate BDNF expression in the brain. Recently, the strategy searching for new compounds, also from natural sources, able to modulate neurotrophin levels, has been increasingly explored. The growing interest in the use of botanicals as modulators of the CNS diseases is proved by the large amount of scientific papers we retrieved and reviewed on this topic (more than one hundred papers for 14 botanicals) (Figure 2). Results critically reviewed and discussed herein emphasize how botanicals modulate BDNF in different pathological conditions affecting the CNS, providing an alternative strategy to the conventional treatment. Indeed, most of the studies demonstrated that treatment with botanicals may prevent and/or normalize the alterations of BDNF caused by experimental handling (Table 2).

An added value of papers dealing with *in vivo* studies is that molecular analyses parallel the investigation of an animal model's behavior after treatment, thus allowing one to draw clear-cut conclusions on the functional outcome associated with the correction of the molecular deficits.

Indeed, despite a variety of different pathological conditions taken into consideration, from mood disorders to Alzheimer's disease and aging up to cerebral vascular damage, the common point is the impairment of cognition. Accordingly, among the behavioral phenotypes, the performance in learning and memory tasks was deeply explored as a common feature of different diseases occurring in the CNS and many botanicals considered have been demonstrated to have cognitive-enhancing properties such as *Bacopa monnieri*, *Coffea arabica*, *Ginkgo biloba*, green tea, olive oil, *Panax ginseng*, *Perilla frutescens*, *Salvia miltiorrhiza*, *Vitis vinifera*, and *Withania somnifera*.

This observation increases the meaning of the results summarized in the present review because the positive effect on a molecular target (BDNF) and on a functional deficit (in cognition) altered in several diseases makes the therapeutic ability of these compounds broad-spectrum.

Another point is that most studies compared the effect of botanicals with the effect obtained from a reference drug, showing similar efficacy. For example, *Bacopa monnieri*, *Crocus sativus*, *Ginkgo biloba*, green tea, *Panax ginseng*, *Perilla frutescens*, and *Rhodiola rosea* exert antidepressant-like effects in different behavioral tests compared to the classical drugs used in therapy. However, most of the clinical studies reported in the present review do not pay attention to the side effects following botanical treatment.

Moreover, even if promising results have been found on *Crocus sativus, Eleutherococcus senticosus* Maxim., *Hypericum perforatum*, *Rhodiola rosea*, *Salvia miltiorrhiza* Bunge, *Vitis vinifera*, and *Withania somnifera* Dunal and BDNF modulation, the number of studies of these botanicals is too low for drawing conclusive results.

The current revision of the literature suggests that several issues need to be considered to draw consistent conclusions. Firstly, considering the complexity of the BDNF system, as briefly described in the introduction, a more refined analysis of the different elements both at transcription and translational levels is mandatory. Indeed, very few studies report the BDNF isoform or the form measured, and, in some cases, the molecular weight of the band examined does not correspond to either the mature or the precursor form.

Secondly, though not negligible, the number of clinical studies is very limited since few clinical trials have been found in literature. Among them, the first was carried out in schizophrenic patients following treatment with *Ginkgo biloba* L. extract [68] and the second was performed in depressed patients treated with *Hypericum perforatum* L. [84], whereas the others were performed in healthy subjects. Although the effect obtained from those studies was relevant, the paucity of clinical trials implies that botanicals discussed in the present review need to be carefully considered for human studies.

Standardization of the extract is an important prerequisite for efficacy of botanicals. Knowledge of the active principles is the first step for an adequate standardization. Results reported in literature show that ginsenosides are mostly responsible for BDNF modulation exerted by *Panax ginseng* C.A. Meyer whereas rhodioloside appears to be the main active principle occurring in *Rhodiola rosea* L. However, for other botanicals, the molecules driving the pharmacological effects are not clearly defined (i.e., *Vitis vinifera* L. or olive oil (*Olea europaea* L.)) and only speculations can be done at this regard. Thus, it is important to underline that most of the studies reviewed herein used standardized extracts, although few papers did not report properly the type of solvent used.

In conclusion, considering the key role of the marker in different pathological conditions affecting the CNS, BDNF may represent an important tool to counteract these conditions, as demonstrated by the studies reported herein. Botanicals may be considered useful candidates to modulate *in vivo* BDNF. If clinical studies confirm this evidence, these natural products may be used for preventing CNS dysfunction or as a useful adjuvant to the pharmacological treatment.

## Figures and Tables

**Table 1:** Brief description of the behavioral test used in the studies reported.

	Test	Protocol	Parameters	Meaning	References
Depression					
Anhedonia	Sucrose consumption/intake	Animals can choose to drink water or 1% sucrose.	Amount of sucrose consumed and preference for water/sucrose	The anhedonic phenotype is characterized by a reduction of sucrose intake/preference.	[25, 26, 64, 99, 100, 102, 103, 119, 123]

Despair	Forced swimming test (FST)	Animals are put in a vessel filled with water.	Latency to floating, swimming time	Despair behavior is associated with shorter latency to float and with less swimming time.	[27, 55, 56, 64, 72, 87, 100–103, 105, 106, 123, 126, 130, 140]
	Tail suspension test (TST)	Animals are suspended by the tail.	Immobility time	Despair is correlated with an increase in the immobility time.	[27, 64, 100, 101, 105, 130]

Anxiety	Open field (OF) test	Animals are free to explore an empty arena.	Time of exploration and number of rearing	Anxiety behavior is correlated with a reduction of exploration and rearing.	[25, 26, 40, 63, 73, 74, 79, 100, 101, 118, 139, 140]
	Shuttle box escape test	Animal can avoid an electric shock by running in the other room of the apparatus.	Number of escapes	Anxiety is characterized by the increased number of escape failures.	[25, 27]
	Elevated plus maze	Animals are free to explore a maze with two open and two close arms.	Time spent in the open arms	The time spent in the open arms is inversely correlated with anxiety.	[73, 104, 106, 140]
	Novelty-induced hyponeophagia (NIH) test	After 48 h food deprivation, animals are put into a cage containing food in the center.	Latency to feeding	Increase latency is associated with an anxious phenotype.	[99, 101]
	Learned helplessness	Animals learn to associate an electric shock with a tone.	Freezing time	Time of freezing is directly correlated with anxiety.	[101]

Cognition	Novel object recognition (NOR) test	Animals must discriminate between a novel (n) and a familiar (F) object.	Time exploring the two objects and NOR index (n − F)/(n + F) + 100	To correctly perform it, the animals must spend more time exploring the novel object. Improvement of cognition is reflected by a higher NOR index, while worsening is reflected by a lower NOR index.	[29, 30, 40, 42, 43, 74, 78]
	Y maze	Animals are put in a maze (Y-shaped), and they must recognize the novel arm (which is closed in the trial phase).	Time exploring the new arm	An increase in time exploring the new arm is an index of a correct cognitive performance.	[29, 78, 113]
	Fear conditioning	Animals learn to associate a cue (context or tone) to an electric shock.	Freezing time when the cue is presented without a shock	Time of freezing is directly correlated with memory.	[29, 33, 77]
	Morris water maze (MWM)	The animals learn to escape onto a hidden platform using this swimming-based model.	Time spent in the target quadrant (where the platform is).	Preserved spatial memory corresponds to increased time in the correct quadrant.	[30, 45, 76, 77, 80, 90, 109, 110, 118, 121, 135, 145]
	Spontaneous alternation test	Animals are placed in the center of a four-arm maze and are free to explore.	Percentage of alternations in the entry of the different arms.	The spontaneous alternation is used as memory task.	[47, 90]
	Radial arm water maze (RAWM)	Animals must find a submerged platform at the end of one of the six arms of the maze, aided by the fixed visually cues on the walls of the room.	Number of errors	Reduction of errors is related to a better cognitive performance.	[52, 91, 139]

**Table 2:** Summary of the experimental conditions employed to investigate the effect of botanical administration on BDNF expression.

Botanicals	Type of studies	Models	mRNA	Protein	References
*Bacopa monnieri* (L.)	*In vitro*	PC12	•		[23, 24]
	*In vivo*	Naïve animals	•	•	[32]
		Chronic stress	•	•	[25, 27]
				•	[26]
		Fear conditioning	•		[33]
		Olfactory bulbectomy	•		[29]
		Scopolamine	•		[30]
			•	•	[31]

*Coffea arabica* L.	*In vitro*	Hippocampal neurons		•	[34, 35]
		Cortical neurons	•		[36]
	*In vivo*	Naïve animals		•	[38–43]
			•		[37]
		Alzheimer's disease	•		[44]
				•	[45]
		Chronic stress		•	[46]
		Obesity (high-fat diet)		•	[47]
		Sleep deprivation	•		[49]
				•	[50–52]
	Clinical	Healthy subjects		*•*	[53]

*Crocus sativus* L.	*In vivo*	Naïve animals	•	•	[55, 56]

*Eleutherococcus senticosus* (Rupr. & Maxim.) Maxim.	*In vitro*	PC12	•		[58]

*Ginkgo biloba* L.	*In vitro*	N2a		•	[59]
		(APP/PS1) primary neurons		•	[60]
		SH-SY5Y		•	[61]
	*In vivo*	Naïve animals		•	[63, 67]
		Alzheimer's disease		•	[60]
		ADHD		•	[61]
		Cerebral ischemia-reperfusion		•	[62]
		Chronic stress		•	[66]
		Haloperidol		•	[65]
		LPS-induced depression		•	[64]
	Clinical	Tardive dyskinesia patients		•	[68]

Green tea (*Camellia sinensis* (L.) Kuntze)	*In vitro*	SH-SY5Y		•	[70]
		Cortical cultures		•	[71]
	*In vivo*	Naïve animals		•	[72–76]
			•		[77]
		Alzheimer's disease		•	[79, 80]
		DYRK1A transgenic mice	•		[78]
		Obesity (high-fat diet)		•	[81]

*Hypericum perforatum* L.	*In vivo*	Chronic stress	•		[82, 83]
	Clinical	Depressed patient		•	[84]

Olive oil (*Olea europaea* L.)	*In vivo*	Naïve animals		•	[87, 88]
			•		[89]
		Alzheimer's disease	•		[91]
		Prenatal stress		•	[90]
	Clinical	Healthy subjects		•	[92]
		Alcoholic patients		•	[93]

*Panax ginseng* C.A. Meyer	*In vitro*	OECs	•	•	[94]
		PC12		•	[98]
		Rat brain slices		•	[96]
		Rat hippocampal neurons		•	[97]
		SCs	•	•	[95]
	*In vivo*	Naïve animals		•	[114, 121, 122]
			•		[120]
		Acute stress	•	•	[108]
			•		[104]
		Alzheimer's disease		•	[115]
		ADHD		•	[61]
		Autoimmune encephalomyelitis		•	[112]
		Cerebral ischemia-reperfusion		•	[116, 117]
			•	•	[118]
		Chronic stress		•	[99, 101–103, 107]
			•		[106]
			•	•	[100]
		Corticosterone	•	•	[105]
		LPS-induced depression	•		[119]
		Scopolamine		•	[110]
			•		[109]
		SAMP8		•	[113]
		Streptozotocin		•	[111]

*Perilla frutescens* (L.) Britton	*In vivo*	Alzheimer's disease		•	[127]
		Chronic stress	•	•	[123]
		Dietary restriction of ALA		•	[124, 125]
		Naïve animals		•	[126]

*Rhodiola rosea* L.	*In vitro*	Mesenchymal stem cells	•	•	[129]
	*In vivo*	LPS-induced depression		•	[130]

*Salvia miltiorrhiza* Bunge	*In vitro*	BM-NSCs		•	[132]
		SH-SY5Y		•	[133]
	*In vivo*	Alzheimer's disease		•	[134]
			•	•	[135]
		Cerebral ischemia-reperfusion		•	[136, 137]
		Streptozotocin and cerebral ischemia-reperfusion		•	[138]

*Vitis vinifera* L.	*In vivo*	Naïve animals		•	[141, 142]
		Acute stress		•	[140]
		Oxidative stress		•	[139]
	Clinical	Healthy subjects		•	[53]

*Withania somnifera* (L.) Dunal	*In vivo*	Hypobaric hypoxia		•	[145]
		Scopolamine	•	•	[144]

Studies which measured the BDNF mRNA or protein levels are indicated with the symbol “•”.
